# Moral experiences of children with medical complexity: A participatory hermeneutic ethnography in Brazil

**DOI:** 10.1177/13674935221112156

**Published:** 2022-09-28

**Authors:** Raíssa Passos dos Santos, Mary Ellen Macdonald, Franco A Carnevale

**Affiliations:** 1 Ingram School of Nursing, 5620 McGill University, Bourcherville, QC, Canada; 2 Faculty of Dentistry, 5620 McGill University, Montreal, QC, Canada; 3 Ingram School of Nursing, 5620 McGill University, Montreal, QC, Canada

**Keywords:** children, childhood, qualitative research

## Abstract

Children with medical complexity have been defined within the literature as chronically ill and medically fragile children with complex care needs. Care for these children raises significant ethical and moral considerations. Therefore, this participatory ethnographic study conducted with eight children and their families aimed to better understand the moral experiences of children with medical complexity, based on views of children as moral agents and capable of understanding and expressing interpretations about their lived experiences. Through our participatory hermeneutical ethnographic research, we were able to shed light on how children with medical complexity express their moral experiences within a complex sociopolitical context, perpetuating dominant outlooks on what is considered a “normal” child. Children with medical complexity described their resistance to these dominant views as they strive to be included in discussions about matters that affect them, reacting to painful medical procedures and treatments, and expressing their concerns about their future aspirations. The knowledge advanced by this study about moral experiences of children with medical complexity can inform understandings of children’s interests based on their own interpretations within complex sociopolitical contexts that value their lives differently.

## Introduction

Children with medical complexity have been defined within the health literature as chronically ill and medically fragile children with complex care needs (Cohen et al., 2011). Care for these children raises significant ethical considerations, and according to the United Nations, “in all actions concerning children, whether undertaken by public or private social welfare institutions, courts of law, administrative authorities, or legislative bodies, the best interests of the child shall be a primary consideration” (UNICEF, 1989, article 3, p. 3). The “best interests” standard is ethically and legally recognized worldwide as a model to guide decisions regarding children (Bubadué et al.,2017). This standard calls for the proportional weighing of the benefits and burdens borne by children, with regard to various treatment options (Bubadué et al., 2017; Carnevale, 2020). However, operationalization of the best interests of children with medical complexity has been problematic in practice (Bubadué et al., 2017; Carnevale, 2020).

The dominant view of children as incapable persons is used to exclude children from participation. In addition, children with medical complexity are frequently described in the health literature as “persons with cognitive impairments,” further complicating inclusion of their views and preferences in decision-making processes (Carnevale et al., 2021; Montreuil et al., 2019). In this context, little is known about children with medical complexity’s best interests and how people responsible for making decisions can elucidate their views in light of their communication differences (Montreuil et al., 2019). Childhood studies' researchers have suggested that understanding children as moral agents (i.e., recognizing children as capable of reasoning and interpreting their lived experiences) is a crucial step toward better including their perspectives and voices in matters that affect them (Montreuil et al., 2018). Children’s moral agency can be understood as “children’s capacity to act deliberately, speak for oneself, and actively reflect on their social worlds, shaping their lives and lives of others” (Montreuil & Carnevale, 2016: p. 510). In this view, children’s communication capacities involve multiple ways of interacting that go beyond verbal communication (Montreuil & Carnevale, 2016).

In some cases, agency is associated with autonomy; however, the similarities and differences between the concepts remain unexplored. Autonomy is commonly construed in terms of a person’s self-determination capacities, which tends to exclude younger children or persons with linguistic or mental function differences. Agency, as defined above, is a more inclusive concept. Moreover, children’s agency is nurtured and expressed in relationships with significant others (Carnevale et al., 2021). Getting others involved in promoting a young person's interests can be beneficial even for young people who are recognized as autonomous (Carnevale et al., 2021).

## Aim

The aim is to examine the experiences of children with medical complexity in relation to their interpretations of their own experiences and how they are shaped by relational, social, historical, and political contexts.

## Methods

A participatory hermeneutic ethnography was used (Montreuil and Carnevale, 2018). This methodology is embedded in Charles Taylor’s hermeneutics which examines human experiences in light of background *social imaginaries* and related *horizons of significance* within a particular social group or context (Montreuil & Carnevale, 2018). Taylor describes *social imaginaries* as “the ways people imagine their social existence, how they fit together with others, how things occur between them and their fellows, the expectations that are normally met, and deeper normative notions and images that underlie these expectations” (Taylor, 2004: p. 23). From this hermeneutical view, people always ascribe meaning to their experiences considering the broader social contexts where human actions are rooted—drawing on *horizons of significance* (Taylor, 1992).

Participatory hermeneutic ethnography allows for an in-depth examination of children’s experiences as morally meaningful (Montreuil & Carnevale, 2018). Hunt and Carnevale (2011) defined these views of experiences from a hermeneutical standpoint as moral experiences. A moral experience “encompasses a person’s sense that values that he or she deems important are being realized or thwarted in everyday life, and this includes a person’s interpretations of a lived encounter or a set of lived encounters that fall on spectrums of right/wrong, good/bad, or just/unjust” (Hunt & Carnevale, 2011: p. 2).

The study received ethical approval from the Institutional Review Board at the university responsible for the study and the local setting where the study was held. Local advisors for this participatory ethnography were children (patients), family members (including siblings), healthcare providers, and managers. Four children, four parents, and four nurses were recruited as study advisors, and subsequent participants were snowball sampled according to the study advisors’ suggestions. All names used are pseudonyms.

Children and families were consulted to decide the best times and places for participant observations and interviews. Three data generation methods were used: participant observation, interviews, and examination of key texts. Documents were analyzed and presented in a different manuscript (Passos dos Santos, 2022). Field notes and audio-recordings from interviews were transcribed into text files. During fieldwork, the researcher’s impressions about the research process, interactions with participants, feelings about fieldwork, and interpretations were shared verbally with children, mothers, and collaborating clinicians. Their insights and thoughts about the researcher’s impressions and interpretations were also sought. Following preliminary interpretations conducted with the research advisors, a descriptive synthesis of each case, including the child and family, was created. Syntheses were then used to identify study exemplars, where similarities and differences were highlighted and contrasted to better understand participants’ lived experiences (Benner, 1994).

### Findings

Eight children with medical complexity participated. Parents, siblings, grandmothers, and healthcare professionals were included in participant observations and informal interviews (totalizing 25 participants). Information about the children and their families is included in Table 1. Healthcare professionals included 4 nurses, 4 physicians, and 2 managers. Children’s clinical conditions included cerebral palsy, Duchenne muscular dystrophy, mucopolysaccharidosis, and Treacher Collins syndrome. Children’s age varied from 4 to 12 years of age. Socioeconomic status of families included low- to middle-income. This sample variation was sought to maximize the diversity of the participant experiences that could be examined.

**Table 1.** Participant details.

Child^1^	Diagnosis	Age	Sex	Primary caregiver	Family members included
Wanda	Mucopolysaccharidosis and Morquio A syndrome	7	F	Mother	Mother and father
Zoe	Cerebral palsy and West syndrome	11	F	Mother	Mother
Peter	Cerebral palsy and severe epilepsy	4	M	Mother	Mother
Steve	Treacher Collins syndrome	5	M	Mother	Mother, father, and sister
Tony	Duchenne muscular dystrophy	11	M	Mother	Mother, father, and sister
Mark	Cerebral palsy	8	M	Father	Father
Tom	Cerebral palsy	6	F	Mother	Mother, grandmother, and 2 brothers
Natasha	Cerebral palsy	12	F	Mother	Mother and sister

^1^All names are pseudonyms.

### Social construction of “the norm”

Virtually every aspect of these children’s lives was surrounded by societal moral outlooks toward the normalization process associated with biomedical aspects of childhood development. These moral outlooks were considered the *hypergood* (Taylor, 1992), that is, the most important good concerning care for these children was to achieve, as much as possible, abilities defined by normal developmental patterns of a child. These pre-established patterns define behaviors and physical skills that children should achieve at certain ages during their path toward adulthood. This broader background context defines “the norm,” which is the socially imagined standard for children’s achievements, and children with medical complexity are presumed to be included.

The background understanding of constantly striving to reach the norm rooted in biomedical views on the healthy child revealed notions around body functionality for children with medical complexity, including expectations of size and walking and using the hands. Based on these understandings, the local *social imaginaries* around healthcare for children with medical complexity encompass the moral order that children ought to align with, to become “normal” healthy children and have a worthwhile quality of life. However, conditions around attempting to reach this norm are poorly addressed and recognized by adults surrounding children with medical complexity. In addition, the interests of healthcare professionals are embedded in these notions, perpetuating and mediating dominant values and practices.

### “The norm” and future expectations about the lives of children

Children’s views on going to the hospital and receiving treatments highlighted their concerns about future expectations of significant others in relation to their lives. One child revealed that by reaching physical patterns such as “*being tall,”* she will consequently be exempted from needing painful medical treatments and going to the hospital. She explained that when she becomes an adult, she would no longer cry and have to agree with adults’ norms because she will finally fulfill others’ expectations. This understanding is embedded in adults’ views that medical treatments can modify children’s physical and clinical realities in the future and help include them in normal patterns defined by “the norm.”

Parents and clinicians considered children “*not yet ready*” to understand their own experiences. A mother described a situation in which her child cried after other children laughed and called him “*ugly*” while playing in a playground. When asked what her response was, the mother explained that she told her child that he is just “*different*” and mentioned that he *“will need psychological support in the years to come to understand what is happening.”* Parents’ actions toward situations like this are based on presumptions about protecting the child because they are “*too young*” to understand. Discourses of the healthcare professionals presented strong views on children being “*too young*” or “*too sick*” to understand their clinical conditions.

### Children’s resistance to the dominant views of “the norm”

These children’s differences were socially constructed as wrong. Societies, therefore, needed to fix these broken children and to turn them into healthy children. Although children are embedded in these social processes, children were not just passive subjects. They expressed multiple forms of resistance to “the norm” frequently under-recognized by people responsible for decisions and actions that affected them. Children’s resistance can be characterized as enactments of their moral agency, expressing their voices toward dominant views on reaching “the norm.” Their expressions about understanding “the norm” have multiple forms, such as crying, silence, non-conformity, and requests for changes in practices.

Children’s resistance was demonstrated through agential expressions of their engagement in their everyday life relationships. Resistance was considered a form of understanding and expression of aspirations, including disagreement with decisions and actions. During medical therapies, children constantly cried, and in doing so, they expressed that this context which privileged constantly trying to reach “the norm,” may be detrimental to their interests. Participant observations of one child provide an illustration:

“During observations, the child shows extreme discomfort. He cries all the time and has several seizures during physical therapy sessions. His mother points out several times that he “doesn’t like physical therapy, but he does it anyway,” meaning that even if he doesn’t like it, the child can perform the activities. The mother’s and healthcare professionals’ statements orbit around the need to perform body movements, such as standing and finding the center of balance of his body. The therapy process aims to perform physical activities that are uncomfortable and cause intense fatigue. The caregivers’ wishes are not compatible with the preferences expressed by the child since they are solely focused on correcting problems, fulfilling tasks, and seeking to achieve developmental milestones” (Peter).

When children’s resistance was not acknowledged and their views were not considered by people responsible for decisions and actions, the dominance of biomedical imperatives of medical procedures can negatively affect children. For instance, one child was submitted to more than 25 nasogastric tube replacements. The child cried and expressed extreme discomfort regarding the repeated re-insertions of the nasogastric tubes. At home, the family, which had minimal economic resources, used a lot of tape to stabilize the nasogastric tube onto the child’s face, increasing her discomfort. Surgery for insertion of a gastrostomy tube took over 4 months to be scheduled. The child constantly demonstrated her resistance to the services she received and her dissatisfaction with practices that subjected her to pain and discomfort. However, her views were not regarded as agential expressions; rather, they were understood as “normal” for what is considered a painful procedure.

Children resisted practices that did not include them in a meaningful manner. One child commented that she once complained to one of her teachers about her teaching methods. According to her, the teacher just “*wrote things on the board*” and did not give her any other activity that she could actively participate in. She then asked the teacher to give her activities in line with her capacities that she could work on during class. The child said, “*I wasn’t going to just sit there*.” Another child described how he had a “*terrible*” year at school when he could not perform the same activities as his classmates, expressing how he felt excluded because “*It was a horrible year! No one wanted to play with me.”* He also explained how he was able to overcome this situation:

- Child: Before, at the beginning of the year, nobody played with me, so it was worse. Now, it’s not so much.

- Researcher: And why did it change?

- Child: Because one day I talked to a psychologist at school, I talked to her and she spoke to my teacher, and my teacher spoke to my classmates. [...] The second-grade teacher had the idea that every day before finishing class, for example, around five o’clock in the afternoon, we would all play games together. [...] Then I started to play because I didn’t play in the second grade. I never, ever played; nobody wanted to play with me.

- Researcher: And playing with others now helps you feel better?

- Child: Yes, because before I didn’t play with anyone (Tony).

### Beyond “the norm”: Life enrichment

Children’s good experiences were expressed mainly through shared moments with people who were important to them. Spending time with siblings and family members, as well as playing with other children, was a part of the life enrichment activities in children’s lives. These experiences are part of everyday moments that are pleasant and morally meaningful to these children.

During participant observation at home, many children expressed their contentment while being around people that they care about. Children demonstrated flexibility regarding frequently challenging situations and their ability to navigate difficult situations and express joy beyond their difficult experiences. One child who expressed constant discomfort also expressed moments of enrichment and satisfaction with her family life at home. The family network was an essential source of support to enrich these children’s lives, providing a different sense of “normality” in which their differences were not highlighted and their interests were understood and recognized. One mother expressed the importance of supporting the positive aspects of her daughter’s life and avoiding negative feelings of helplessness and pity that can cause harm by making her feel sad about being a burden to her family. In turn, the child always responds with smiles and demonstrates her happiness in being with her family. Optimism within the child’s social context fosters her positive experiences, which are constantly expressed during her daily routines, at school, and in medical encounters. According to the mother, other family members and friends treat her “*just like other children*” without differences due to her medical complexity.

Children expressed their gratification in sharing moments with other children and participating in activities that foster good aspects of their experiences. When their teachers could understand and listen to what was important to them, schools were a source of enrichment. For instance, one child was attending a school where her teacher could create activities in line with her capacities. During classroom observations, the child performed activities with the teacher, such as brush painting, paper cutting, and collages, incessantly smiling, and showing satisfaction. During a school break, she smiled when meeting other children. Some children asked to “*take her for a walk*” and pushed the wheelchair around. Children shared their views when they made friends at school and were assigned activities aligned with their capacities. In these situations, the school can be a joyful space for children to disconnect from painful procedures and other difficult realities. When asked about what was good in his life, one child always referred to activities related to school. He mentioned being thrilled about his new friends at school and the activities he was involved with. His mother added that since he stopped going to the hospital so often and started school, he was a much “*happier*” child and had many new friends.

Children’s participation in decisions that affect them was a significant aspect that fostered their good experiences. When families and healthcare professionals recognized children’s perspectives about medical treatments, practices that are more respectful could be achieved by including the child’s interests in the decisional process, for instance:

“In the consultation with the orthopedist, the child entered the room with her mother. The mother spoke with the physician and asked about the case’s severity and whether surgery was really necessary. The physician asked the child if she was having trouble opening her legs or in pain at any time, who confirmed that she did not feel pain, could still open her legs, and that she thought she did not need another surgery. The mother confirmed she could perform her life activities “normally.” The physician then confirmed that if she does not feel pain and could maintain her “normal” functions, the surgery could be postponed until it was really necessary. I later asked the child if she was happy with the decision, and she confirmed that she was still worried about needing to be operated on in the future, although she felt relieved not having to go through another complex medical procedure” (Natasha).

When clinicians are more engaged with children’s preferences, they can integrate social contexts surrounding them with medical complexity into their practices, possibly avoid harmful procedures. For instance, when one nurse was highly engaged with a child’s suffering, she could understand the child’s crying beyond the biomedical imperatives of performing medical procedures. She expressed that the situation was “*absurd,”* and she was interested not only in “*covering her shift,”* but she also wanted “*to solve people’s problems.”* This healthcare professional carried out actions that encouraged her colleagues to manage the surgery process that could prevent multiple nasogastric tube replacements.

### Relational perspectives of voices and interests of children with medical complexity

For many children, mothers and sisters had important roles as their interlocutors. The interlocutor can be defined as someone able to understand the child’s expressions and concerns and is ultimately someone with whom the child is confident sharing their experiences. For one child, the presence of unfamiliar people became a threat since his initial socialization involved being surrounded by those who performed painful procedures. During participant observation, the researcher talked to his sister, and she explained to the child the questions that were asked. The child responded when his sister repeated the questions to him. Hence, as someone he trusted, his sister’s presence made his experiences more positive, helping him disclose his views and aspirations. In another situation, a child’s brothers were able to understand if she expressed pain or discomfort and would then inform other people about what was happening.

The relational perspectives of these children were illustrated in the complex relationship between mothers’ voices and child’s interests. Mothers’ interpretations of crying as expressions can inform healthcare professionals about their children’s needs. When these interpretations are not considered by people responsible for decisions and actions, the children’s interests may not be fully represented, sometimes resulting in disengaged practices and the child’s discomfort. For example, the following situation was observed:

“The child presented episodes of intense crying after a physical therapy session at home. The mother took the child to the pediatric emergency department. After physical examination, the residents and their preceptor concluded that the child did not have any other significant symptoms and asked the mother to return the next morning. According to the mother, her child did not sleep and “howled” in pain when his diaper was being changed. The next day, the mother returned, and, once again, the physicians on duty did not identify other symptoms and reported that the child had an “unidentified/unknown reason to cry.” According to the mother, the physicians expressed that she was exaggerating because crying is something expected for a child with “neurological impairments.” The mother returned to the hospital after three days of roundtrips, and the physician on duty then ordered an X-ray, which revealed a bilateral fracture of the femur and hip dislocation” (Peter).

Some children described how they should comply with their mothers in relation to their medical treatments, even though their views about the importance of medical treatment were not always in line with their mothers’ views. When asked about the importance of going through a corrective hip surgery, one child mentioned that she was afraid and “*hoped not to need it,”* although she would consent to the procedure “*if her mother thinks she needs to.”* Another child mentioned that she “*wanted to grow taller because her mother wanted her to,*” and therefore, she was consenting to the medical treatment.

Children demonstrated their desire to protect their mothers from suffering, avoiding expressions of sadness and satisfying their mothers’ wishes to support them. Sometimes this desire seemed to express a child’s caring conscience for their loved ones. As one child explained, when he talked about his illness, his mother cried, and he did not like seeing his mother cry. During participant observations, the child constantly emphasized to his mother that he is “*not ill*” and that “*some new medication will make him “better*.” The mother said that her child “*gives me so much strength, a strength that I as a mother should give him instead.”*

## Discussion

Within this participatory hermeneutic ethnography, we have demonstrated how voices of children with medical complexity are deeply rooted in the relational, social, historical, and political contexts surrounding them. People responsible for decisions and actions have a significant impact on how we understand the experiences of these children. The voices of children were expressed through the voices of their interlocutors, who are people who have significant knowledge about their lives. The mothers’ voices have weight in decisions that will affect children’s lives, from the socially constructed views of women as responsible for the child’s care, and from the relational interests of children, which are oriented by what matters to significant others. In addition, the social context in which these children are embedded has significant implications for how they experience their everyday lives. Socially, historically, and politically constructed notions about childhood and normal child development affect how children with medical complexity experience their lives, both positively and negatively. Importantly, children are not merely passive spectators of what is happening in their surroundings: they are active agents who express their resistance to the dominant views about normal development and future expectations about their lives. The actions of people who are responsible for treatment decisions and their recognition and inclusion of children’s views have meaningful impacts on their experiences.

The social construction of “the norm” that permeated the moral experiences of children in this study is consistent with what Gibson et al. (2015) referred to as dominant social views, in which children are considered “normal” and “good” concerning their functional abilities (Gibson et al., 2015). In line with this dominant view, studies about people with disabilities are primarily focused on biological and physiological differences, resulting in problems around “normalization” and how the bodies of people with disabilities need to be “fixed” to function productively (Fadyl et al., 2020). Within this view, societal and medical understandings of children with medical complexity are rooted in biomedical notions where children are primarily regarded in terms of their failures (i.e., what they “can’t” do) and their complex clinical conditions. In this context, future expectations and notions of a “healthy child” are permeated in the experiences and encounters with healthcare services of children in this study.

Historically, the field of pediatrics was constructed around notions of development and the trajectory from childhood to adulthood (Gibson et al., 2015). Developmental researchers have established patterns to help clinicians predict and inhibit threats for children becoming productive adults (James & James, 2017). In the study’s setting, clinicians and parents planned healthcare decisions and actions to reach the dominant view of a “healthy life” as defined in views on good pediatric healthcare. In order to reach a “healthy life,” children were expected to match standardized developmental stages. Parallel with this view, Gibson et al. (2015) highlighted that, in traditional pediatrics, deviations from normal development measures are considered problematic, and interventions to re-insert children into normal patterns must be implemented. In a critical examination of the goal of rehabilitation and disability, Phelan (2011) described how constant attempts to achieve goals in line with the social constructions of “the normal” could result in frustration for children when they are not included in decision-making processes.

Beyond the social and historical implications of “the norm,” by including children in the research process, we could demonstrate their own understandings of *social imaginaries* of “reaching the norm.” Many children stated their resistance to “the norm,” meaning that they were not always in accordance with what adults expected for their lives. In many situations, people responsible for decisions and actions did not recognize children as moral agents who can thoughtfully participate in decisions about matters that affect their lives. This corresponds with another study with children in a mental health setting where participants demonstrated their dissatisfaction with practices, and their experiences were widely discounted by staff members (Montreuil et al., 2018).

Some children described their positive experiences regarding inclusion in a social group, such as participating in activities with a group in their school. These positive experiences shared by children emphasized the importance of participating in communal activities as an experience that indicates good aspects of “normality” in their lives. These views are similar to data from a scoping review on the experiences of children with medical complexity that showed children’s discourses on good aspects related to being included in a group and participating in school activities (Santos et al., 2020). In a study with children with disabilities, Connors and Stalker (2007) described how children expressed feelings of happiness associated with being part of a group and performing the same activities as their school peers. These situations perpetuated the “sameness” of children’s experiences and prevented negative feelings (Connors and Stalker, 2007).

This study demonstrated the complex dynamism of children’s interdependence and relationships. Some children expressed their preferences in relation to what matters to others. For instance, some children mentioned that they agreed with health treatments to make their mothers happy. Spyrou (2019) highlighted that acknowledging this relational basis in children’s experiences can help expand understandings of children’s voices and perspectives. As highlighted by Carnevale et al. (2017), a more in-depth understanding of children’s interests is more likely to be achieved by acknowledging relational perspectives involved in the beliefs and values of both children and parents. While children are somewhat dependent on people responsible for making decisions on their behalf (parents or legal representatives), they are not deprived of significant moral views (Carnevale et al., 2017). Adopting research and care approaches that include these relational perspectives could contribute to a better understanding of children’s interests and preferences.

### Study limitations

As a one-setting study that examined local Brazilian perspectives, this study has limitations regarding the multiple sociocultural contexts in which children with medical complexity can live. Studies conducted in different social-political contexts could reveal new nuances and perspectives of children’s experiences that were not explored in this study. Therefore, further research is recommended to conduct in-depth analyses within other settings to improve knowledge of the experiences of children with medical complexity in various contexts.

### Implications for practice

This study describes how understanding children’s moral experiences with medical complexity can shed light on how to understand these children’s best interests. Norms and legal standards that guide treatment decisions state that healthcare professionals should promote children’s best interests based on a presumption of children’s best interests as the course of action that will optimize the balance of benefits in relation to burdens for the child. This presumption is rooted in social, historical, and cultural perspectives of childhood and children with medical complexity. As a result, some of the hardships that children are navigating are imposed in the name of benevolence behind dominant notions of protection. These hardships are also amplified in the name of treatments, which characterize the differences of children with medical complexity as pathologies, meaning problems to be fixed by biomedical-centered health interventions. Thus, children with medical complexity ought to achieve certain goals and reach health patterns to be healthy children, despite possible discomforts and children’s resistance.

## Conclusion

This study provides an account of the moral experiences of children with medical complexity in Brazil. It describes how the multiple *social imaginaries* on reaching “the norm,” future expectations, and maternal care, interact and influence their experiences and how children express their resistance to practices that conflict with their interests. Using inclusive ways of understanding children’s moral experiences and being attentive to their multiple forms of communication can help reimagine children’s actual lived interests and lead healthcare professionals and people responsible for decisions and actions to more ethical practices to help children with medical complexity thrive. Finally, using the moral experiences’ framework helped reveal less recognized interests and how children experience certain interests. This understanding helps us shed new light on whether some medical treatments are worthwhile in light of their adverse effects on children’s interests, including playing, pleasure, and love.

## References

[bibr1-13674935221112156] Benner P (1994) The tradition and skill of interpretive phenomenology in studying health, illness, and caring practices. In: Interpretive Phenomenology: Embodiment, Caring, and Ethics in Health and, pp. 99–127.

[bibr2-13674935221112156] Bubadué RDM Cabral IE Carnevale F , et al. (2017) Análise normativa sobre a voz da criança na legislação brasileira de proteção à infância. Revista Gaúcha de Enfermagem, 37. 10.1590/1983-1447.2016.04.58018 28198942

[bibr3-13674935221112156] Carnevale FA Collin Vézina D Macdonald ME , et al. (2021) Childhood Ethics: An ontological advancement for childhood studies. Children & Society 35(1): 110–124.

[bibr4-13674935221112156] Carnevale FA (2020) A “Thick” conception of children’s voices: A hermeneutical framework for childhood research. International Journal of Qualitative Methods 19: 767.

[bibr5-13674935221112156] Carnevale FA Teachman G Bogossian A (2017) A relational ethics framework for advancing practice with children with complex health care needs and their parents. Comprehensive Child and Adolescent Nursing 40(4): 268–284. 29161173 10.1080/24694193.2017.1373162

[bibr6-13674935221112156] Cohen E Kuo DZ Agrawal R , et al. (2011) Children with medical complexity: an emerging population for clinical and research initiatives. Pediatrics 127(3): 529–538. 21339266 10.1542/peds.2010-0910 PMC3387912

[bibr7-13674935221112156] Connors C Stalker K (2007) Children’s experiences of disability: Pointers to a social model of childhood disability. Disability & Society 22(1): 19–33.

[bibr8-13674935221112156] Fadyl JK Teachman G Hamdani Y (2020) Problematizing ‘productive citizenship’within rehabilitation services: insights from three studies. Disability and Rehabilitation 42(20): 2959–2966. 30829075 10.1080/09638288.2019.1573935

[bibr9-13674935221112156] Hamdani Y (2015) Rethinking “Normal Development” in Children’s Rehabilitation In: Rethinking Rehabilitation. CRC Press, pp. 90–103.

[bibr10-13674935221112156] Hunt MR Carnevale FA (2011) Moral experience: a framework for bioethics research. Journal of Medical Ethics 37(11): 658–662. 21515876 10.1136/jme.2010.039008

[bibr11-13674935221112156] James A James A (2017) Constructing Childhood: Theory, Policy and Social Practice. Macmillan International Higher Education.

[bibr12-13674935221112156] Montreuil M Teachman G Carnevale FA (2019) Recognizing the Voices of All Children, Including Those with “Cognitive Impairments,” in Research In: Research Involving Participants with Cognitive Disability and Difference 135-148. Oxford University Press.

[bibr13-13674935221112156] Montreuil M Noronha C Floriani N , et al. (2018) Children’s moral agency: An interdisciplinary scoping review. Journal of Childhood Studies 17–30.

[bibr14-13674935221112156] Montreuil M Carnevale FA (2016) A concept analysis of children’s agency within the health literature. Journal of Child Health Care 20(4): 503–511. 26666263 10.1177/1367493515620914

[bibr15-13674935221112156] Montreuil M Carnevale FA (2018) Participatory hermeneutic ethnography: A methodological framework for health ethics research with children. Qualitative Health Research 28(7): 1135–1144. 29542396 10.1177/1049732318757489

[bibr16-13674935221112156] Phelan SK (2011) Constructions of disability: A call for critical reflexivity in occupational therapy. Canadian Journal of Occupational Therapy 78(3): 164–172. 10.2182/cjot.2011.78.3.4 21699010

[bibr17-13674935221112156] Raíssa Passos Dos Santos (2022) Moral Experiences of Children with Medical Complexity: Contributions from a Study in Brazil. Raíssa Passos Dos Santos.

[bibr18-13674935221112156] Santos RP Macdonald ME Carnevale F (2020) A Scoping Review of the Moral Experiences of Children With Medical Complexity in Brazil. Revista brasileira de enfermagem 73(2): 20190268. 10.1590/0034-7167-2019-0268 32236380

[bibr19-13674935221112156] Spyrou S (2019) An ontological turn for childhood studies? Children & Society 33(4): 316–323.

[bibr20-13674935221112156] Taylor C (2004) Modern Social Imaginaries. Duke University Press.

[bibr21-13674935221112156] Taylor C (1992) Sources of the Self: The Making of the Modern Identity. Harvard University Press.

[bibr22-13674935221112156] UNICEF (1989) Convention on the Rights of the Child. OHCHR.

